# Factors associated with cesarean delivery rates: a single-institution experience

**DOI:** 10.1186/s40748-017-0047-z

**Published:** 2017-04-21

**Authors:** Spencer McClelland, Naomi Gorfinkle, Alan A. Arslan, Maria Teresa Benedetto-Anzai, Teresa Cheon, Yuzuru Anzai

**Affiliations:** 1 NYU Langone Medical Center, Department of Obstetrics and Gynecology, 800 2nd Avenue, Suite 815, New York, NY 10017 USA; 2 Johns Hopkins University School of Medicine, 4 South Broadway, Baltimore, MD 21231 USA; 3 NYU Langone Medical Center, Division of Epidemiology, Departments of Obstetrics and Gynecology, Environmental Medicine, and Population Health, 650 First Ave, Rm. 532, New York, NY 10016 USA

**Keywords:** Cesarean delivery, Cesarean section, Reduction, Forceps, Provider volume

## Abstract

**Background:**

The aim of this study was to identify factors associated with variability in Cesarean delivery (CD) rates amongst providers at a single institution.

**Methods:**

A retrospective cohort analysis was carried out on all births at NYU Langone Medical Center from 2005–2013. Data was collected for subjects and linked to diagnosis codes for singleton and twin deliveries. Descriptive characteristics were generated for all deliveries, and inferential analysis was performed including multiple covariates for singleton deliveries in the 2010–2013 cohort, including both univariate and multivariate regression analyses to identify factors associated with higher CD rates.

**Results:**

37,692 deliveries were identified at our institution during the study period, performed by 88 unique providers. The mean CD rate was 29.6%, with a range for individual physicians from 9.9% to 75.6%. In multivariate regression analysis, CD rate was directly correlated with average patient age, physician male gender, proportion of high-risk deliveries, and Maternal-Fetal Medicine specialty, and it was inversely correlated with total number of deliveries by physician and forceps delivery rate. There was no significant difference in CD rates between group and solo practices. Within the same group practice, each member’s CD rate was strongly correlated with the average CD rate of the group.

**Conclusion:**

Our study demonstrates the wide range of CD rates for providers practicing within the same institution and reiterates the association of CD rates with patient age, high-risk pregnancy, and provider volume. Among operative vaginal deliveries, forceps delivery rate was associated with lower CD rates whereas vacuum delivery rate was not. Despite these findings, practice patterns within individual practices appear to contribute significantly to the wide range of CD rates.

## Background

Cesarean delivery (CD) is the most commonly performed major surgery in the United States, accounting for approximately one third of all deliveries [1]. The CD rate had been increasing steadily in recent years, although the national rate has remained stable for the past three years [2]. Compared to vaginal deliveries, CDs are associated with higher maternal morbidity and mortality and can lead to significant complications in future pregnancies [3–5]. While CDs carry clear maternal risks, many studies have failed to show any clear benefits, particularly with regard to neonatal outcomes that would favor CD over vaginal delivery. In light of this evidence, there is growing concern regarding the rising CD rates in United States.

CD rates vary significantly by geographic location and between hospitals, with reported hospital rates ranging from 7.1 to 69.9% [6]. A number of factors have been identified that contribute to this variability, such as regional differences in operative vaginal delivery rates, practice models, (e.g., use of laborists), and medico-legal environments [6–8]. Studies have also been published demonstrating a marked variation in CD rates between providers within an institution, which argues against regional differences being the only—or even the dominant—factor determining CD rates [9, 10].

Recent publications by the Eunice Kennedy Shriver National Institute of Child Health and Human Development (NICHD), Society for Maternal-Fetal Medicine, and American College of Obstetricians and Gynecologists have cast attention on the rise in CD rates, with a goal of modifying practices and factors that lead to non-indicated CDs. Although certain conditions requiring CD are non-modifiable (e.g. complete placenta previa, cord prolapse) and others are strongly influenced by standards of care and common practice (e.g. malpresentation), these represent a relatively small proportion of the cited indications for CD in the U.S. The two most common indications for CD are labor arrest and non-reassuring fetal status. The criteria for diagnosing these conditions are often subjective, and variation in rates of these diagnoses are believed to contribute to the significant variation in CD rates [3–5]. The above publications have also identified use of operative vaginal delivery as a factor influencing CD rates, with the continued observation that the waning use of operative vaginal deliveries has resulted in decreasing opportunities for training, creating a vicious cycle that will result in fewer and fewer physicians becoming proficient in operative vaginal deliveries.

Among the major academic medical centers in New York City for 2011, CD rates ranged from 25.3 to 38.8% [11]. During this period, the published CD rate at NYU Langone Medical Center was 27.7%, placing our institution at the lower end of the spectrum. In this study, we analyzed the CD rates within our single institution from 2005 to 2013 for all providers, with the goal of demonstrating our intra-hospital range in CD rates and identifying factors that may have contributed to variation.

## Methods

This retrospective study of deliveries at NYU Langone Medical Center from 2005 to 2013 was approved and received exempt status by the Institutional Review Board. Virtually all patients either had private insurance or were self-pay. Deliveries were almost always performed by private attending physicians who independently dictate management decisions, including the mode of delivery.

Data in this study were extracted from several sources. For deliveries from 2010 to 2013, the data was extracted from the Clinical Database Resource Manager of the University HealthSystem Consortium (UHC). UHC is a data aggregator that collects information from over 200 University and University–affiliated hospitals. It stores clinical information such as diagnosis and procedure codes, as well as information on patient demographics, complications, and readmissions. It has been validated in prior studies with regard to its fidelity to other available databases and has been used in many publications [12]. We extracted data for subjects linked to the diagnosis codes V270 (Singleton Deliveries) and V272 (Twin deliveries). We excluded multiple gestations higher than twins because these accounted for very few deliveries and were almost always delivered by Cesarean section.

Extracted data included patients’ age, race, mode of delivery (Normal Vaginal Delivery, Cesarean Delivery, Forceps Delivery, Vacuum Delivery), delivering physician code, and presence or absence of multiple medical and obstetric co-morbidities. The modes of delivery were stratified by using codes for the delivering physicians, and CD rates and operative vaginal delivery rates for the different physicians were calculated. We linked the physician codes to classify gender, place of residency training, year of completion of residency, practice, and type of practice model.

In our institution, the physicians are either solo practitioners, who are on call on their own and cover the majority of deliveries for their own patients, or belong to group practices, where responsibility for patients is shared and call coverage is split between the members of the practice. Nineteen physicians are solo practitioners and 69 physicians are part of group practices (9 groups in total).

For the deliveries from 2005–2009, we obtained data from NYU Langone Medical Center’s administrative records. This database contains information for all deliveries including mode of delivery(CD, spontaneous vaginal delivery or operative vaginal delivery), physician code, and whether the patient received a blood transfusion. This database is more limited than the UHC database, and does not include data such as patient age or race, number of gestation, or medical and obstetric co-morbidities.

For this reason, our analysis was performed in two parts. Descriptive statistics were analyzed for both cohorts, inclusive of all deliveries from 2005–2013. These included total number of deliveries (by type), mean rates of different delivery types for the hospital, and CD rates for different providers. Mean hospital length of stay, cost of hospitalization per patient, and blood transfusion rates were also calculated for each physician.

Inferential analysis was performed on singleton deliveries from the 2010–2013 cohort only, to more accurately analyze the influence of different factors on the CD rates of individual providers. Univariate analysis was performed using Chi-square test for categorical variables, and ANOVA test for continuous variables. Correlation analysis was carried out to calculate Pearson correlation coefficients (r), followed by multivariate regression analysis to identify variables that were independently associated with CD rates. Multivariate regression analysis was carried out using a stepwise backward elimination approach to identify the most significant variables influencing CD rates. Variables included in the initial multivariate model were: average patient age, physician years since completion of residency, physician gender, total number of deliveries, forceps rate, vacuum rate, specialty (MFM, non-MFM), provider (specific provider or practice), proportion of high-risk deliveries by provider, and practice model (solo, group) [13]. The statistical analyses were performed using IBM SPSS Statistics for Windows, Version 21.0. (Armonk, NY: IBM Corp.).

The intraclass correlation coefficient (ICC) was calculated using two-way random effects model. The ICC is commonly used to quantify the degree to which individuals (in our case, OB providers) resemble each other in terms of a quantitative trait (in our case, singleton CD rate). High ICCs suggest a strong within-provider correlation (or high reliability) of CD rate from year to year, while small ICCs indicate that observations are similar across providers, suggesting no group or clustering effect [14]. As a rule of thumb, ICC values less than 0.5 are indicative of poor reliability, values between 0.5 and 0.75 indicate moderate reliability, values between 0.75 and 0.9 indicate good reliability, and values greater than 0.90 indicate excellent reliability [15].

Providers with fewer than 30 deliveries per year were excluded from this study. When physicians perform very few deliveries, their apparent CD rates may not be an accurate representation of their true CD rate, as a result of a small sample size. They may appear to have an artificially high or low CD rate, which would skew our statistical analysis.

A final key assumption for our analysis was the use of a physician’s total singleton CD rate as the dependent variable, rather than the primary CD rate, low-risk CD rate, or other rates. To support this decision, we performed preliminary correlation analyses of individual physicians’ CD rates, which are discussed in the Results section below.

## Results

From 2005–2013, 88 providers were included in our analysis, with a total of 37,692 deliveries performed between them at our institution. 57 providers were female and 31 were male. The providers trained at a variety of programs and completed residency from 1975 up until 2013. Fourteen providers were identified as MFM subspecialists, and 74 providers were identified as general Obstetrics and Gynecology specialists. The mean patient age was 32.9 +/− 1.4 years. 67% of patients identified as White, 5.0% as Black, 13.7% as Asian, and 13.4% other.

From 2005–2013, the mean CD rate was 29.6%, with an individual range by provider by year of 9.9 to 75.6%. From 2010–2013, there were 385 twin deliveries, with a mean twin CD rate of 73.2%. The mean operative vaginal delivery rate was 5.2%, with an individual provider range of 0 to 22.4%. The mean forceps rate was 1.8% and the mean vacuum rate was 3.4% (Table 1). For the entire hospital, the mean length of stay per patient was 2.8 days, and the mean cost per patient of hospitalization was $6498.73.

**Table 1:** Delivery statistics by year, 2005-2013

Year	All deliveries	Cesarean deliveries	Cesarean delivery rate, mean %	Cesarean delivery rate, range	Twin deliveries	Twin Cesarean delivery rate	Operative vaginal delivery rate	Forceps vaginal delivery rate	Vacuum vaginal delivery rate
2005	3627	1111	30.6%	10.8–49.4%	N/A	N/A	6.0%	N/A	N/A
2006	3720	1148	30.9%	14.8–55.2%	N/A	N/A	4.9%	N/A	N/A
2007	4318	1375	31.8%	16.4–62.2%	N/A	N/A	4.8%	N/A	N/A
2008	4477	1389	31.0%	11.8–65.6%	N/A	N/A	4.8%	N/A	N/A
2009	4332	1270	29.3%	15.1–55.3%	N/A	N/A	4.0%	N/A	N/A
2010	4373	1254	28.7%	12.7–50.8%	103	69.9%	4.2%	1.6%	2.6%
2011	4331	1245	28.7%	11.5–66.0%	98	74.5%	4.6%	1.6%	3.6%
2012	4019	1113	27.7%	10.2–75.6%	108	75.0%	5.6%	1.9%	4.5%
2013	4495	1233	27.4%	9.9–65.8%	76	73.7%	5.5%	2.2%	2.7%
Total	37692	11138	29.6%	9.9–75.6%	385	73.2%	5.2%	1.8%	3.4%

Singleton CD rate was very strongly correlated with overall CD rate (*r* = 0.993), singleton primary CD rate (*r* = 0.941), and singleton CD rate excluding non-modifiable indications such as malpresentation, abnormal placentation, and cord prolapse (*r* = 0.984). Twin deliveries were an extremely small proportion of the overall deliveries and did not contribute significantly to any provider’s CD rate. Given the above, twins were excluded from our inferential analysis, and singleton CD rate was used for all subsequent descriptive and inferential statistical analyses.

To account for factors known to influence CD rates, we separated the deliveries into low-risk and high-risk deliveries. “High-risk” deliveries were those associated with the following diagnoses, as used in multiple similar database-derived studies: advanced maternal age, malpresentation, previous Cesarean delivery, placenta previa, preeclampsia and hypertension, obesity, preterm delivery, thyroid disease, asthma, fetal-placental problems, amniotic infection, maternal fever, and cord prolapse [16–20]. Providers’ individual low-risk and high-risk CD singleton CD rates were strongly correlated (*r* = 0.62, *p* <0.0001) [21]. High-risk deliveries constitute the majority of deliveries at our institution at 55.9%, with a range from 30.0 to 77.4% by provider. In light of these facts, we included high-risk deliveries in our analyses, in order to accurately reflect the case-mix at our institution, and to ensure our findings would be generalizable to and pertinent to similar academic institutions.

Providers who were identified as MFM specialists had a significantly higher CD rate than other providers (41.8% vs 29.9%, *p* <0.0001). Male providers had a higher mean CD rate than female providers (33.6% vs 29.9%, *p* = 0.002).

With regard to practice model, the mean CD rate was 33.0% and 31.0% for solo and group practices, and this difference was not statistically significant (solo vs group, *p* = 0.13). Within the group practices, the CD rates ranged by practice from 10.2 to 65.6%. The CD rates of the individual providers within those practices ranged from 9.9 to 75.6%. Among the group practices, the average CD rate of each physician was strongly correlated with their practice’s CD rate (*r* = 0.72, *p* < 0.0001).

The intraclass correlation coefficient (ICC) for the CD rates among the providers was 0.76 (*p* = 0.0001), suggesting that there was good reliability within providers’ personal rates over time, and a much higher rate of variability between providers. In other words, each provider’s CD rate remained relatively constant during the period we analyzed, and the wide range of CD rates observed in our institution was therefore primarily a function of differences between providers.

On univariate correlation analysis, singleton CD rate was directly correlated with the following provider characteristics: mean patient age, years since completion of residency, and proportion of high-risk deliveries. It was inversely correlated with the following provider characteristics: total number of deliveries, operative vaginal delivery rate, and forceps rate. Singleton CD rate was also directly correlated with mean hospital length of stay and cost per patient, but not with the rate of blood transfusions (Table 2).

**Table 2:** Pearson correlations between selected characteristics and singleton CD rate

	Singleton CD rate	Patient age	Total deliveries, n	Singleton operative vaginal delivery rate	Forceps rate	Vacuum rate	Blood transfusion rate	Hospital length of stay	Cost per patient	Years since graduation	Specialty (MFM)	Proportion of high-risk deliveries
Singleton CD rate	1	0.30^b^	−0.39^b^	−0.24^b^	−0.30^b^	−0.04	0.13	0.45^b^	0.41^b^	0.30^b^	0.34^b^	0.50^b^
Patient age		1	−0.28^b^	−0.09	0.03	−0.03	0.11	0.07	0.09	−0.16^a^	−0.03	0.57^b^
Total deliveries, n			1	−0.02	0.40^b^	0.05	−0.21^b^	−0.24^b^	−0.26^b^	0.07	−0.13	−0.29^b^
Singleton operative vaginal delivery rate				1	0.61^b^	0.50^b^	0.10	0.01	0.03	−0.02	−0.06	−0.05
Forceps rate					1	−0.04	−0.07	−0.17^a^	−0.14	0.07	−0.13	−0.12
Vacuum rate						1	0.09	0.17^a^	0.12	0.01	−0.03	−0.07
Blood transfusion rate							1	0.19^a^	0.33^b^	0.10	0.16^a^	0.17^a^
Hospital length of stay								1	0.79^b^	0.21^b^	0.38^b^	0.27^b^
Cost per patient									1	0.20^b^	0.38^b^	0.37^b^
Years since graduation										1	0.31^b^	0.10
Specialty (MFM)											1	0.34^b^
Proportion of high-risk deliveries												1

^a^Correlation is significant at the 0.05 level (2-tailed)

^b^Correlation is significant at the 0.01 level (2-tailed)

In the multivariate regression analysis, singleton CD rate remained positively associated with mean patient age, physician male gender, proportion of high-risk deliveries, and MFM specialty, and negatively associated with forceps rate and total number of deliveries (Table 3).

**Table 3:** Multivariate linear regression modeling^a^ of factors influencing singleton CD rate

Factor	Standardized Beta	*P*-value
Physician gender (males vs. females)	0.369	0.0001
Patient age	0.301	0.0004
Proportion of high-risk deliveries	0.210	0.01
Specialty (MFM vs. non-MFM)	0.147	0.027
Forceps rate	−0.240	0.0003
Total number of deliveries	−0.209	0.003

^a^Multivariate linear regression model using step-wise backward elimination procedure. The variables included in the initial multivariate model included: average patient age, years since completion of residency, physician gender (male, female), total number of deliveries, forceps rate, vacuum rate, specialty (MFM, non-MFM), provider (specific provider or practice), practice model (solo, group), and proportion of high-risk deliveries (percent). The following factors were removed by the model since they met the criteria for removal (*p* > 0.05) in order of removal (practice model, vacuum rate, provider, and years since completion of residency)

## Discussion

CD is associated with both higher maternal mortality and higher morbidity, namely hemorrhage, infectious morbidity, and the need for emergent hysterectomy, and these risks are even higher when comparing post-labor CD to scheduled CD. CD also brings with it the potential for complications in future pregnancies, such as the need for repeat CDs, or the possibility of abnormal placentation (previa, accreta). It is also associated with increased costs to both patients and hospitals. Despite these downsides, there is not demonstrated improvement in neonatal outcomes [22, 23]. Many private insurance companies provide higher reimbursement rates for CD than for either spontaneous vaginal delivery or operative vaginal delivery, both of which can require significantly more investment of time and skill on behalf of an individual provider. And although CD rates are a national quality measure when applied to hospitals, the same is not true of individual physicians, who are neither rewarded for low CD rates nor penalized for high CD rates [24].

It is widely known, even to the general public, that CD rates differ among practitioners. However, there have been few publications regarding the variation of CD rates among individual physicians practicing in the same setting [25, 26]. Therefore, it is important to analyze the factors affecting individual variation in CD rates in order to better address the national goal of reducing the CD rate, particularly non-indicated Cesarean deliveries.

There exists a wide range of CD rates among the providers and practices in our study. The ICC for the CD rates among the individual providers was 0.76, suggesting good inter-observer reproducibility, i.e., the rates within individual providers are more similar than the rates between individual providers. Within group practices, the CD rates of the individual providers were strongly correlated with the mean practice rate. This may suggest that there are factors within individual practices that encourage or discourage trends in CD, such as similarities in prenatal counseling, management of labor, thresholds for CD, and operative delivery skills, as well as similarity in patient populations and risk factors.

We identified several factors that were independently associated with higher CD rates. Increasing patient age is a known risk factor for CD, and providers with higher average patient ages had higher CD rates [27].

Providers who had higher numbers of deliveries had lower CD rates. This is consistent with prior publications regarding the effect of provider volume on clinical outcomes in other fields, and it also supports the findings of Clapp et al., who demonstrated a similar phenomenon with regard to provider volume and CD rates [28, 29]. This finding is plausible insofar as physicians who perform more deliveries may be more comfortable managing labor and have different thresholds for performing CDs compared to providers who practice less frequently. As the above authors point out, this also represents a potentially modifiable factor for providers and hospitals seeking to reduce CD rates.

Male providers had higher CD rates than female providers, and this remained true on multivariate regression analysis. This may be due to the specific providers within our institution and may not be generalizable in other practice settings.

Providers with higher operative vaginal delivery rates had lower CD rates. This observation is consistent with prior studies and with ACOG recommendations regarding the importance of training in operative vaginal delivery [30]. It is also in agreement with the recent publication by Fitzwater et al. which showed increased CD rate in nulliparous women in the second stage of labor in conjunction with the decreasing operative vaginal delivery rate [31]. It is important to note that higher forceps rates were significantly associated with lower CD rates, while higher vacuum rates were not. In keeping with this finding, there seems to be a consensus among experts in operative vaginal delivery that the risk of failure is higher with vacuum than with forceps deliveries [32]. This relationship has implications for resident education, as training residents in vacuum deliveries may not be as effective in lowering CD rates as would training them in forceps deliveries. Given the decreasing frequency with which forceps are practiced, it may be worth considering limiting the teaching of forceps deliveries to those physicians who will practice obstetrics in the future, as recently suggested by Barth [33].

Providers with higher proportions of high-risk deliveries had higher CD rates, which is an expected finding. Providers who identified as MFM subspecialists had higher CD rates, and this was true on both univariate and multivariate analysis, and was independent of the effect of proportion of high-risk deliveries. While it is possible that the higher CD rates for MFM providers are related to management of high-risk pregnancies, several points argue against this explanation. First, the existing literature is clear that very few high-risk conditions warrant choosing CD as mode of delivery preferentially over induction and vaginal delivery at the point at which delivery is indicated [4, 5]. Second, many of the most common high-risk conditions (particularly preeclampsia and PPROM) are managed by both generalist and MFM providers, and even when MFM providers act as consultants, the delivering provider is often the patient’s primary physician (i.e. original generalist provider), which should reduce the observed difference. Lastly, high-risk conditions that mandate CD over vaginal delivery (maternal contraindications to vaginal delivery, fetal malformations, etc.) are rare and unlikely to explain the differential we observed in CD rates. We therefore do not have a strong explanation for this finding, other than individual provider practice.

One additional finding in our study was the role of practice model. Solo providers are ones who manage their patients in their own practice and deliver the majority of their own patients. Group practices share prenatal care responsibilities and divide labor and delivery call responsibilities based on shifts. Based on these categories, we found that there was no statistically significant difference in CD rates between solo providers and group practices. This runs counter to some speculation, particularly in the lay press, that solo providers are prone to higher CD rates than group practices because of fatigue and a desire to expedite delivery of their patients in order to limit their time in the hospital. In addition, the high correlation of CD rates among physicians in the same group practices suggests a possible effect of similar labor management style and protocols regarding CD.

To the best of our knowledge, this is one of few studies examining the characteristics of individual physicians and their influence on CD rates. There are several strengths to this study. First, there is a large sample size, both in terms of number of providers and number of deliveries. Second, the single-institution nature allows us to analyze physician factors in a homogenous practicing environment, potentially correcting for variations in hospital factors (infrastructure, ancillary services, medico-legal context, etc.) that may lead to variations between institutions. Lastly, we were able to analyze many important covariates, including controlling for high-risk conditions so as to demonstrate the influence of other factors in a mixed low-risk and high-risk population.

This study is not without limitations. While we were able to control for obesity and preterm delivery, the retrospective nature limited our ability to collect certain covariates like body mass index, parity, and gestational age at delivery in more detail. Also, as we were using two separate databases, we were only able to perform inferential statistics on one half of our total cohort. Additionally, we are not able to account for the effect of differences in prenatal care by the different providers, particularly with regard to significant risk factors for CD, such as maternal weight gain.

Despite these limitations, we believe our findings add to national and international conversations regarding CD by shedding light on certain potentially modifiable factors. The range of CD rates observed in the providers in a single institution, both among MFM subspecialists and generalists, was wide. Although we were able to identify several factors associated with higher CD rates, these are unlikely to explain the extremely high rates observed in some providers. Our findings reinforce the influence of patient age on CD risk, as well as the more newly identified effect of provider volume on CD rates. Our findings also emphasize once again the importance of skills in forceps-assisted vaginal deliveries as a means of reducing CD rates, over and above the utility of vacuum-assisted vaginal deliveries, a finding that continues to have important implications for the training of residents.

## Conclusions

There remains great variability between providers practicing at the same institution with regard to CD rates. This variability is related to many factors, including patient variables like age and high-risk pregnancy, but also provider variables, such as volume of practice, rate of forceps deliveries, and MFM specialty. These findings reinforce the need for addressing efforts to reduce CD rates at the institution level by focusing on potentially modifiable provider factors that may drive higher CD rates.

## Abbreviations

CD: Cesarean delivery
ICC: Intraclass correlation coefficient
MFM: Maternal-Fetal Medicine
